# The Validation and Improvement of a Food Literacy Behavior Checklist for Food Literacy Programs

**DOI:** 10.3390/ijerph182413282

**Published:** 2021-12-16

**Authors:** Ellen Paynter, Andrea Begley, Lucy M. Butcher, Satvinder S. Dhaliwal

**Affiliations:** 1 School of Population Health, Curtin University, Bentley 6102, Australia; ellen.paynter@curtin.edu.au; 2 Foodbank Western Australia, Perth 6105, Australia; lucy.butcher@foodbankwa.org.au; 3 Curtin Health Innovation Research Institute, Faculty of Health Sciences, Curtin University, Bentley 6102, Australia; s.dhaliwal@curtin.edu.au; 4 Duke-NUS Medical School, National University of Singapore, Singapore 169857, Singapore; 5 Department of Radiation Oncology, Sir Charles Gairdner Hospital, Nedlands 6009, Australia; 6 Institute for Research in Molecular Medicine, Universiti Sains Malaysia, Minden 11800, Malaysia

**Keywords:** food literacy, validity, adult, low income, measurement, scale, checklist

## Abstract

Food literacy is a multidimensional construct required to achieve diet quality. The Food Sensations^®^ for Adults (FSA) program aims to improve the food literacy of low to middle-income adults living in Western Australia and is funded by the Western Australian Department of Health. The original published behavior checklist used to measure change in food literacy has been revised based on experience of the facilitators and the iterative development of the program. This research sought to assess the validity and reliability of the improved food literacy behavior checklist. A total of 1,359 participants completed the checklist over an 18-month period. Content, face, and construct validity were considered in the re-development of the checklist. An exploratory factor analysis of the checklist identified three factors: (1) Plan and Manage, (2) Selection, and (3) Preparation. Cronbach’s alpha coefficients of 0.883, 0.760, and 0.868 were found for each of the three factors respectively. These coefficients indicated good internal consistency and were higher than those found in the original checklist analysis. An external validation was undertaken with the original food literacy behavior checklist, and a strong positive relationship between the two tools was found. In addition to being used to evaluate FSA, this revised and extensively validated tool could provide guidance to others evaluating similar food literacy programs and contribute to international measurement research.

## 1. Introduction

Nutrition education programs are designed to target a community’s food skills and behaviors to improve dietary intake and reduce diet-related diseases. These programs often focus on cooking skills or combine aspects of nutrition education and cooking in recognition of the broad range of food skills people require [1,2]. The knowledge, skills, and behaviors required to achieve diet quality are conceptualized as ‘food literacy’. Vidgen and Gallegos [3] have provided the most widely cited definition of food literacy, which was based on empirical research in Australia. Specifically, they defined food literacy as “a collection of interrelated knowledge, skills and behaviors required to plan, manage, select, prepare and eat foods to meet needs and to determine food intake” [3]. The implementation of food literacy behaviors has been shown to result in better diet quality; conversely, low food literacy and low confidence in the use of food literacy skills are considered barriers to healthy eating [4].

An increased focus on improving food literacy has resulted in programs being funded. This focus has led to research being conducted to produce both general and program evaluation tools. The conceptualization of food literacy and of what constitutes a food-literate person has resulted in tools that consider a range of indicators, including the complexity of knowledge, self-efficacy or confidence, attitude, and behavior indicators [3,5,6,7]. Food literacy is a multidimensional construct; illustrating this, international experts reviewing 229 items to achieve a consensus informing an item pool of 119 items [8]. Measurement tool development is therefore challenging, and there is currently no accepted gold standard reference tool for comparison [9]. Different purposes, such as population food literacy skills monitoring and food literacy program evaluation, mean that it is unlikely that any one measurement tool could suit all purposes [10]. Population monitoring and specific program evaluation will require standardized and objective, or context specific tools. In relation to the purpose of program evaluation, tools need to be purposively aligned with programs’ objectives and funders’ priorities, and short in duration to reduce the burden placed on participants in communities with lower levels of English language proficiency. One of the major limitations to establishing program effectiveness is the use of non-validated evaluation tools [1].

Food literacy measurement tools that have been published thus far vary in terms of scales, number of questions, and sample target groups. Despite the greater delivery of cooking and food literacy interventions in the last decade [1], the research and evaluation of food literacy measurement is still evolving [8]. For example, it was only recently that a scoping review provided comparative evidence of evaluation tool validity and reliability [11]. This scoping review identified 12 tools, with five that explicitly measured food literacy. These 12 tools had been developed to measure food literacy for four distinct purposes: to measure food literacy with general tools, food literacy strategy indicator tools, or food literacy specific program evaluation tools, or to measure food literacy as part of a broader study [11]. None of the tools measured all the food literacy components defined by Vidgen and Gallegos [3]. Further, only eight of the tools reported the reliability of their measures using Cronbach’s alpha, with internal consistency ranging from 0.76–0.95, and there was heterogeneity in the characteristics and size of the sample populations [11]. Consequently, valid tools need to continue to be developed, including new short general or evaluation tools, and existing tools need to be improved.

Food Sensations^®^ for Adults (FSA) is a four-week, ten-hour food literacy program developed by Foodbank Western Australia (WA) and funded by the WA Department of Health with the aim of improving food literacy in low to middle-income participants. FSA contains four core modules, which are taught over the first three (of four) sessions: (1) healthy eating; (2) label reading and food selection; (3) meal planning and budgeting; and 4) food preparation and cooking. In the fourth and final session, an additional optional module is selected by participants to facilitate the contextualization of food literacy to the needs of specific groups, such as prisoners, retirees, or parents. Each week, half of the session is dedicated to education and in the other half participants cook and eat together. A food literacy behavior checklist (FLBC) was developed to assess the service level outcomes required by the funder, and the questions included in the checklist were originally sourced from the Expanded Food and Nutrition Education Program’s (EFNEP’s) food behavior checklist [12,13] and other published program evaluations [14,15]. The EFNEP’s food behavior checklist has been in use since 1997 and there is strong evidence of the validity and reliability of the 10 core questions [12]. The Version 1 FLBC was designed to capture the self-reported frequency of behaviors in the previous month and used pre- and post-program results to assess change. The content, face, and construct validity of the FSA’s FLBC were assessed and the first version (Version 1) of the tool was used for program evaluation from 2016 to 2018 [16]. New contract funding, a request for a new evaluation plan from the funder, and further program development including curriculum iterations required a review to be undertaken of the Version 1 tool. A 15-item second version of the FLBC (Version 2) was used to evaluate the program from July 2018 to December 2019. This study aimed to assess the validity and reliability of the revised FLBC and evaluate the measurement properties of the new checklist against the previous checklist to produce a revised tool for evaluation.

## 2. Materials and Methods

This study consisted of phases including questionnaire development, statistical analysis of scale testing, and validation to produce and test Version 2 of the FLBC. Data were used from a cross-sectional study using pre-program data collected from adult participants attending a food literacy program. The item development, scale testing, and validation of Version 1 of the FLBC have been reported previously [16].

### 2.1. Version 2 Questionnaire Development

Scale development processes were used to direct the validation of the original tool [17], including processes for nutrition education evaluation [18] and best practice recommendations [19]. The construct validity and internal consistency reliability analysis identified three factors in the Version 1 FLBC: (1) *Plan and manage*, (2) *Selection*, and (3) *Preparation*. These three factors matched three of the four domains in the Australian food literacy model [3]. The original factor analysis showed that 12 of the 14 questions loaded above the cut-off for one of these factors, and these 12 questions were used to produce factor scores to demonstrate the impact [20] and evaluate the program outcomes between 2016 to June 2018.

The experience program facilitators gained since 2016 program delivery, further program development including curriculum iterations, and the awarding of new contract funding for continued program delivery from July 2018 required that a review be undertaken of the FLBC. The WA Department of Health requested a new evaluation plan with the awarding of new contract funding. The lesson plans were reviewed by the program facilitators and the emphases on program message content and reinforcement were then assessed against the questions in the FLBC by the evaluation team to establish content validity. The new session objectives were realigned with the questions as part of the content validity analysis and the review of the published literature. The questions for Version 1 and Version 2 are compared in Table 1.

As Version 1 contained two questions related to the use of food labels that produced very similar results, one of these questions was removed (i.e., the following question was removed, “Use other parts of food label to make food choices*”*). At the start of the program, the “Thaw meat at room temperature” behavior was reported as being infrequent. After the lesson plan review mid-2018, the program facilitation team changed their focus for food safety content delivery in lesson plans. The “*Check the use-by date on foods*” question was added as a food safety behavior measure. The increased emphasis of the FSA program for the new contract funding on developing self-efficacy for change (confidence) resulted in two additional questions being added, that is, “*Felt confident selecting low cost healthy foods?*” and “*Felt confident making changes in your food choices?*”. The Likert scale was expanded to include the following ratings: *Never*, *Rarely*, *Sometimes*, *Most of the time*, and *Always*. The questions were selected based on their alignment with the session objectives (content validity) and tested for face validity in early program delivery with participants and facilitators. 

The face validity of a draft of Version 2 of the FLBC was tested with diverse participants in three FSA programs that were running at the end of the first contract period (*n* = 22). Brief cognitive interviews were conducted with participants in the final program week. Participants were asked to complete Version 2 FLBC and prompted to discuss how they found the item wording and comprehension [21]. The same demographic questions used with FLBC Version 1 were incorporated with FLBC Version 2. These included sex, age, postcode, household structure, education, employment, birth in Australia, and identification as Aboriginal or Torres Strait Islander.

### 2.2. Sample

Participants attending FSA between July 2018 and December 2019 were invited to complete the pre-program questionnaire including the FLBC before commencing the program. FSA programs were run in metropolitan and regional areas of WA. FSA delivery is targeted at low to middle income participants; however, it is open to all Western Australians to enroll and high-income participants are not refused enrolment.

### 2.3. Analysis

The socioeconomic index (SEI) [22] was calculated using residence postcodes, as outlined previously [16]. The responses to the food literacy behavior questions containing the heading ‘*How often have you done the following actions in the last month?*’ were coded 1–5 for *Never*, *Rarely*, *Sometimes*, *Mostly*, and *Always*, respectively. As the “*Run out of money for food*” was negatively worded, the responses were reverse coded before the analysis. The analyses were conducted using SPSS (IBM) Version 25.

The factor analysis employed two methods: scree plots and the pattern of factor loadings. The Kaiser–Myer–Olkin (KMO) value was calculated to ensure an adequate sample size, and Bartlett’s test of sphericity was conducted to determine whether the dataset was suitable for factor analysis. The exploratory factor analysis with generalized least squares extraction identified three factors as guided by the scree plots. The scree plots showed the relative importance of each factor and were used as a guide for extraction. Varimax rotation was used, as factors were predicted to be independent. A factor loading cut-off of 0.4 was set based on accepted convention, and our previous analyses [16]. To assess the reliability of each factor, Cronbach’s alpha was used; a value > 0.7 indicated good internal consistency [23]. This reflects the degree to which the questions in the checklist co-varied relative to their sum score.

To investigate how the questions clustered within and between factors, two-dimensional scatter plots of factor loadings in which the factor was indicated were created. As access to the raw data from Version 1 was available to the research team, convergent validation of the updated food literacy behavior tool was facilitated by comparison with the original FLBC (Version 1) [16]. Scatter plots, Pearson’s correlation coefficients, and significance values were calculated to investigate the type and strength of the relationship between the original and improved food literacy behavior tools. The Cronbach’s alphas, KMO values, and Bartlett’s test of sphericity results for each of the two tool versions were compared.

### 2.4. Ethics

The Human Research Ethics Committee at Curtin University granted ethics approval for the program evaluation processes and tools (RDHS-52-16). The program facilitators outlined the purpose of the research to participants both verbally and via a written information sheet. Informed written consent was obtained upon completion of the checklist and cognitive interviews.

## 3. Results

### 3.1. Cognitive Interview

Program participants were able to distinguish between the five-point Likert scale. Based on the test results, minor wording changes were made to Version 2 of the FLBC. For a summary of the questions included in the first and second versions of the FLBC, see Table 1. According to the Flesch–Kincaid readability test, the final FLBC had a reading-grade level of 78.8, which indicated that the material uses plain English and was rated as fairly easy to read.

### 3.2. Response Rate

All attendees of the FSA program between July 2018 and December 2019 were invited to fill in the questionnaire at the start of the program. Of the 1612 attendees invited to fill in the questionnaire, 1359 consented to participate in the study and completed the questionnaire at the start of the program.

### 3.3. Participant Demographics

Of the questionnaire participants, 70.6% identified as female (see Table 2). The ages of the participants ranged; the largest proportion (23.5%) fell in the 26–35-year-old group. In the present study, 8.3% of participants identified as Aboriginal or Torres Strait Islander.

In this study, 61.0% of the participants reported that they were born in Australia. Almost one-half (44.5%) of participants lived in low SEI areas. Approximately one-third (37.2%) of participants lived in a couple with children or with family. Over one-third (35.3%) of participants reported that their highest level of education was a certificate, diploma, or a trade. One-quarter of participants (25.2%) reported that they had completed some high school. Over one-third (35.7%) of participants reported that they were currently unable to work, undertaking house duties, or were retired. Just under one-third (30.8%) of participants reported that they were currently unemployed or unable to work.

### 3.4. A Factor Analysis and the Reliability of the New Food Literacy Behavior Tool

A KMO value of 0.922 indicated that the sample size was suitable for a factor analysis. Bartlett’s test of sphericity *p*-value < 0.0001 indicated that the data were suitable for a data reduction technique, such as factor analysis. Using a factor loading cut-off of 0.4, 13 of the 15 factors loaded onto one of the three food literacy behavior factors (see Table 3).

**Figure 1:**
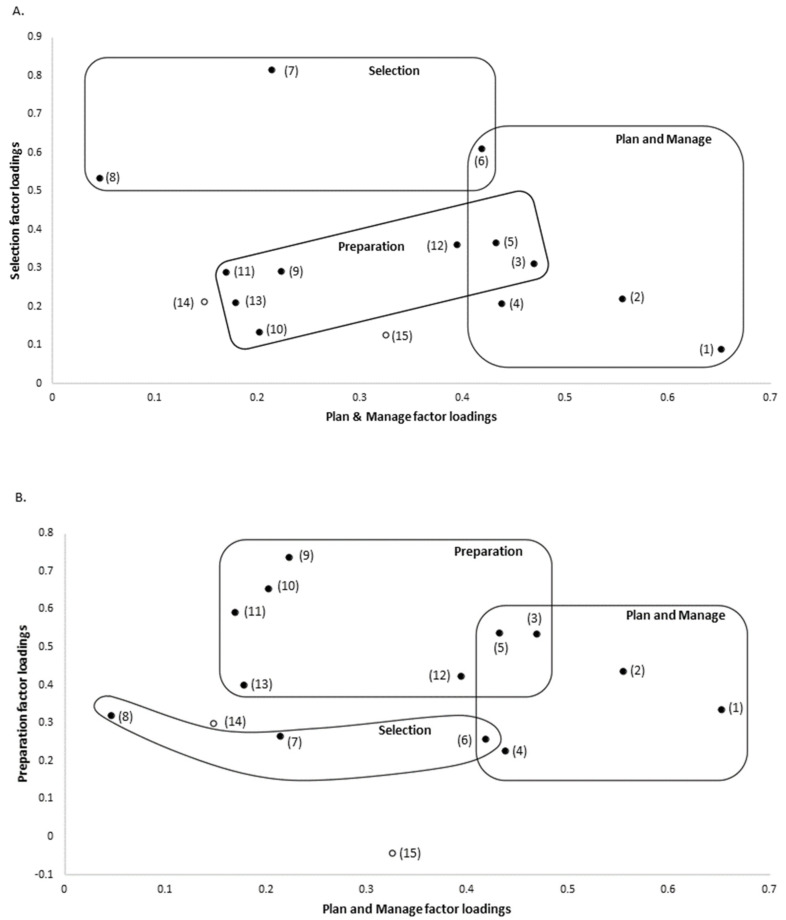
Two-dimensional plots of the three food literacy behavior factors. (**A**) Plan and Manage (x axis) and Selection (y axis) factor loadings. (**B**) Plan and Manage (x axis) and Preparation (y axis) factor loadings. The three food literacy behavior factors are indicated on the graphs, with questions scoring ≥ 0.4 indicated. Unfilled points indicate questions that did not significantly load on any factor.

These three factors were named Plan and Manage, Selection, and Preparation. Two questions (i.e., “*Check the use by date on foods*” and “*Run out of money for food*”) had low factor loadings for all three factors and thus did not significantly load onto any factor. Cronbach’s alpha coefficients of 0.883, 0.760, and 0.868 for Plan and Manage, Selection, and Preparation, respectively, indicated good internal consistency within each factor. The leftmost column of Table 2 shows the questions relating to food literacy behaviors, which were analyzed in relation to the generation of the food literacy behavior tool. Factor loadings for the questions are shown in the columns under each factor; those in bold type indicate factor loading scores of ≥0.4, and Cronbach’s alpha coefficients appear in parentheses directly below each food literacy behavior factor. Figure 1 shows two-dimensional plots of factor loadings that indicate which of the questions loaded onto each of the three factors.

### 3.5. Comparison to Version 1 FLBC Factor Loadings

**Figure 2:**
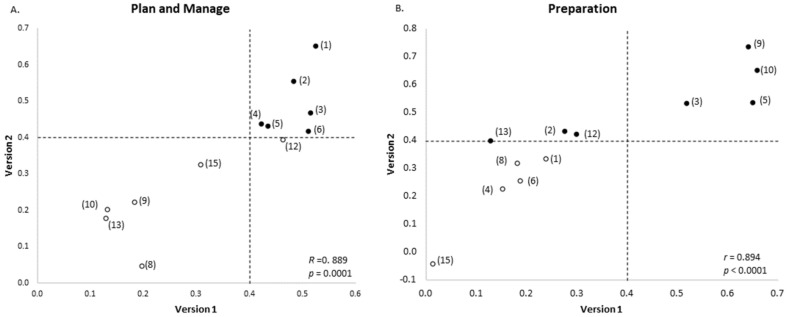
Comparison of factor loadings in food literacy behavior tools of those questions common to both questionnaires. (**A**) Plan and manage factor; (**B**) Preparation factor. x axis: original food literacy behavior tool based on Version 1 of the questionnaire. y axis: revised food literacy behavior tool from Version 2 of questionnaire. The dashed line indicates 0.4 cut-off used. Filled in points indicate factor loading ≥ 0.4 in analysis of questionnaire Version 2.

The exploratory factor analysis of the two datasets yielded similar, but non-identical results. This was due to changes made to the Version 2 FLBC (Figure 1). Specifically, one question was removed from the initial checklist and three questions were added to the new checklist. More participants completed the questionnaire in this analysis of the Version 2 FLBC than the previous analysis of the Version 1 (i.e., 1359 compared to 1012). In both cases, the three factors identified broadly covered the same food literacy domains, and the factors were named the same in both versions: *Plan and Manage*; *Selection*, and *Preparation*. All of the *Plan and Manage* questions included were consistent between the analyses, except that the “*Think about healthy choices when deciding what to eat*” question no longer met the loading threshold at 0.394 and was thus not included in the new factor (see Figure 2A).

The new Preparation factor contained the same four questions as the original analysis, plus an additional four questions, that is, “*Use a nutrition information panel to make food choices,* “*Plan meals to include all food groups*,” “*Think about healthy choices when deciding what to eat*,” and “*Felt confident in making changes in your food choices”* (see Figure 2). The last-mentioned question represents a new addition to the FLBC. The aforementioned three questions scored only 0.129, 0.277, and 0.300, respectively, in the original analysis.

The Selection factor did not share any questions with the previous analysis. It contained one new question (i.e., “*Felt confident to select low cost healthy foods*”) and two questions that were also present in the original FLBC (i.e., “*Compare prices of foods to find the best prices on healthy foods*” and “*Felt confident about managing money to buy healthy food*”). In the original factor analysis, the “*Compare prices of foods to find the best prices on healthy foods*” question fell just short of the loading cut-off at 0.366; however, the “*Felt confident about managing money to buy healthy food*” was well below the threshold at 0.187. Of the two questions in the original Selection factor, one question was removed from the subsequent FLBC and the second “*Use a nutrition information panel to make food choices*” question did not meet the cut-off at 0.213.

Scatterplots are not shown for Selection, as this factor did not contain any common questions with factor scores ≥ 0.4 in the analyses of either version of the FLBC. The “*Run out of money for food*” question did not meet the threshold for any of the three factors in the analysis of the original FLBC or the updated checklist. The new “*Check the use by date on foods*” question did not meet the threshold for any of the factors in the analysis.

Plots of the factor loadings for each question in the original and improved food literacy behavior tools revealed a positive relationship. Pearson’s correlation showed that these positive relationships were significant for Plan and Manage (*r* = 0.889, *p* = 0.0001) and Preparation (*r* = 0.894, *p* < 0.0001). Bartlett’s test of sphericity yielded a *p* < 0.0001 in both the original and updated analyses. The KMO value in the analysis of the updated food literacy behaviors tool was 0.922, which was slightly higher than the score of 0.892 in the original analysis. Cronbach’s alpha values for each of the three factors were higher for the Version 2 FLBC factors compared with the original factors. The Plan and Manage alpha coefficient increased from 0.790 to 0.883, the Selection alpha coefficient from 0.756 to 0.760, and the Preparation alpha coefficient from 0.812 to 0.868.

## 4. Discussion

This research adopted an approach in which multiple phases of item development, scale measurement, and validation were applied. We aimed to improve the measure of food literacy behaviors targeted in an adult food literacy program to produce an evaluation or short general tool for use at multiple points of time. The dataset for the present study captured a higher proportion of the target group, adults from low socioeconomic areas, than the previous validation study [16]. This has implications for the English literacy levels required tool completion. The revised FLBC has several strengths, including being grounded in the most widely cited definition of food literacy [3], which covers a range of food literacy behavior domains reflecting theoretical conceptualizations. Other strengths of the revised FLBC include the fact that validity and reliability assessments were used in its development, the sample size, and the range of demographic characteristics of the participants used in the scale testing.

There was strong consistency between the two FLBC versions. Improvements to the original FLBC, the large number of participants included in the analysis, and the increased proportion of the target audience likely contributed to the increase in the internal consistency and the reliability of the revised food literacy behavior tool. The “*Run out of money for food*” question did not meet the factor cut-off in either version of the tool. This was the only question to measure both planning and management skills and assess food security in the food literacy behavior tool; thus, it makes sense that participants’ answers to this question did not cluster with answers to other questions in the questionnaire. Similarly, the “*Check the use by date on foods*” question was the only food safety question, and it did not significantly load on any of the three factors. Notably, both these questions would have been included, or close to meeting the criteria for inclusion, if a cut-off of 0.3 for loadings had been used. The inclusion and/or revision of these three questions can be reviewed for future FLBC developments.

Several questions loaded above 0.4 on a different factor in the Version 2 FLBC. The “*Think about healthy choices when deciding what to eat*” question changed from Plan and Manage in Version 1 to Preparation in Version 2. The “*Plan meals to include all food groups*” question loaded onto both Preparation and Plan and Manage in Version 1, but only loaded onto the latter factor in Version 2. The “*Felt confident about managing money to buy healthy food*” question loaded onto Selection in Version 2 in addition to Plan and Manage in Version 1. The “*Use a nutrition information panel to make food choices*” question changed from loading onto Selection in Version 1 to Preparation in Version 2. The “*Compare prices of foods to find the best prices on heathy foods*” question did not make the 0.4 cut-off in Version 1 of the tool, but loaded onto Selection in Version 2 of the tool. In most instances, the questions that changed the factors on which they loaded onto in versions 1 or 2 could reasonably fit in either of the food literacy domain reflected by the factors. For example, the “*Think about healthy choices when deciding what to eat*” question could be considered part of both the Plan and Manage and Preparation domains of food literacy [3].

Our scale development processes were similar in their systematic approach to other studies, including those in the United States [24], Italy [25], Switzerland [26], Netherlands [27], and Taiwan [28]. Notably, while the aims of all these research studies are similar, there are differences in the constructs employed by these tools. This may reflect the different uses and priorities assigned to the psychological constructs in the tools and food literacy domains. No food literacy measurement tool aligns fully with the four domains of food literacy [11] conceptualized by Vidgen and Gallegos [3] due to their different purposes. Many tools have a focus on the preparation and cooking knowledge and skills domain [29,30]. Despite comprising only 13 questions, the Version 1 FLBC tool was one of the most comprehensive food literacy behavior tools available, as the factors represent three of the four domains of food literacy defined by Vidgen and Gallegos [3]. Many of the Version 2 FLBC items are the same or similar to items achieving high consensus in a recently published content validity international food literacy survey [8].

We used the advantage of having a large sample size for reliability analysis testing to produce this type of tool. Compared to the samples used to examine the development of Versions 1 and 2 of the FLBC, other development, scale measurement, and validation studies used smaller samples [24]. For example, the Canadian food skills questionnaire was developed using 165 participants [9], and the EFNEP’s food behavior checklist for the Cooking Matters program was validated using samples between 12–95 participants [31]. The higher proportion of female attendees in the FSA program is consistent with other food literacy programs [9,26,29,30]. This may reflect the traditional role women play in providing food for the family and the fact that women are more likely to attend education programs.

When developing an evaluation tool, it is critical that consideration is given to the burden placed on participants, the time available to administer the tool, and the tool being fit for purpose. Version 2 of the FLBC contained a modest number of questions in comparison to up to 49 items in other food literacy tools being produced [11], and the FSA FLBC was short and specific to the lesson objectives. Other reported short tools contain a similar number of questions, and while such tools are feasible for use, they only report unidimensionally with one factor [26]. More extensive scales are not suitable for program evaluation due to the number of items, and are often only tested on highly educated volunteers [9,25]. With the exception of the Selection factor, which was still considered statistically acceptable, the reliability of the factors within this improved food literacy behavior tool are comparable to other tools [11]. Now that it has been validated, this improved food literacy behavior tool can be used in future evaluation and research on FSA or other similar food literacy behavior programs; for example, it could be used to investigate the effects or effectiveness of programs.

There are limitations that should be acknowledged in developing scales based on behavioral constructs. Next steps would include—further scale evaluation, including a temporal stability assessment. Temporal stability is also described as test-retest reliability, or to use the same tool on two occasions with the aim of producing the same results [32] The FLBC is specific to the FSA program evaluation and thus needs to be tested for use with different program content and other types of participants. This evaluation tool does not produce a factor based on the eating domain of food literacy. This is because the focus of the evaluation questions was on program content and was based on the lesson objectives. As discussed above, in real-world program delivery to low-income participants, any such evaluation tool needs to be able to be completed in a short period and be suitable for a range of literacy levels.

## 5. Conclusions

Further evidence is needed to confirm that investment in food literacy programs that include low-income groups is warranted for improving dietary intakes and meeting community requirements. It is critical that comprehensive efforts continue to be made to improve the validity and reliability of tools that accurately measure program effectiveness.

## Figures and Tables

**Table 1:** Comparison of questions included in the Food Literacy Behaviors Checklist (Versions 1 and 2).

	Version 1	Version 2
1	Plan meals ahead of time	Plan meals ahead of time
2	Plan meals to include all food groups	Plan meals to include all food groups
3	Think about healthy choices when deciding what to eat	Think about healthy choices when deciding what to eat
4	Make a list before you go shopping	Make a list before you go shopping
5	Run out of money for food	Run out of money for food
6	Felt confident about managing money to buy healthy food	Felt confident managing your money to buy healthy food
7	Compare prices of foods to find the best prices on healthy foods	Compare prices to select low cost healthy foods
8	Use a nutrition information panel to make food choices	Use a nutrition information panel to make food choices
9	Use other parts of the food label to make food choices	-
10	Cook meals at home using healthy ingredients	Cook meals at home using healthy ingredients
11	Felt confident about cooking a variety of healthy meals	Felt confident cooking a variety of healthy meals
12	Try a new recipe	Try a new recipe at home
13	Change recipes to make them healthier	Change recipes to make them healthier
14	Thaw meat at room temperature	-
15	-	Check the use by date on foods
16	-	Felt confident selecting low cost healthy foods?
17	-	Felt confident making changes in your food choices?

**Table 2:** Demographic characteristics of program attendees completing the start of program questionnaire.

Characteristic		Participant Number (%)
Sex (*n* = 1356)	Female	958 (70.6%)
	Male	394 (29.1%)
	Other	4 (0.3%)
Age (*n* = 1306)	18–25 y	47 (11.3%)
	26–35 y	307 (23.5%)
	36–45 y	243 (18.6%)
	46–55 y	175 (13.4%)
	56–65 y	158 (12.1%)
	66 and over	276 (21.1%)
Socioeconomic Index ^1^ (*n* = 1165)	Low	518 (44.5%)
	Middle	354 (30.4%)
	High	291 (25.0%)
Household Structure (*n* = 1316)	Couple with child/children and/or other family	489 (37.2%)
	Couple without child/children	284 (21.6%)
	Single person	248 (18.8%)
	Single parent with child/children	130 (10.0%)
	Group/supported accommodation	165 (12.5%)
Education Level (*n* = 1302)	Certificate/Diploma/Trade	460 (35.3%)
	Some high school	328 (25.2%)
	Bachelor or higher	277 (21.3%)
	Finished high school	237 (18.2%)
Employment Status (*n* = 1310)	Away from work/house duties/retired	468 (35.7%)
	Unemployed or unable to work	403 (30.8%)
	Part-time or casual	286 (21.8%)
	Full-time	153 (11.7%)
Born in Australia (*n* = 1322)		807 (61.0%)
Identify as Aboriginal or Torres Strait Islander (*n* = 1282)		107 (8.3%)

^1^ Derived from postcodes’ socioeconomic indexes for areas [22].

**Table 3:** Revised food literacy behavior checklist, factors, and factor loadings (*n* = 1359). The three columns alongside show the scores of the items for each of the three factors. The scores above the threshold of 0.4 were accepted in the factors (shown in bold font) and Cronbach’s alpha analyses, indicated in parentheses following each factor title.

How Often Have You Done the Following Actions in the Last Month? (Available Answers: Never, Sometimes, Most of the Time, Always)	Factors and Factor Loadings		
	Plan and Manage (0.883)	Selection (0.760)	Preparation (0.868)
(1) Plan meals ahead of time	**0.652**	0.089	0.335
(2) Plan meals to include all food groups	**0.555**	0.219	**0.435**
(3) Cook meals at home using healthy ingredients	**0.469**	0.312	**0.534**
(4) Make a list before you go shopping	**0.438**	0.208	0.227
(5) Felt confident about cooking a variety of healthy meals	**0.432**	0.365	**0.537**
(6) Felt confident about managing money to buy healthy food	**0.418**	**0.611**	0.257
(7) Felt confident to select low cost healthy foods ^1^	0.214	**0.816**	0.264
(8) Compare prices of foods to find the best prices on healthy foods	0.046	**0.534**	0.320
(9) Change recipes to make them healthier	0.223	0.292	**0.737**
(10) Try a new recipe at home	0.202	0.133	**0.654**
(11) Felt confident in making changes in your food choices ^1^	0.169	0.288	**0.592**
(12) Think about healthy choices when deciding what to eat	0.394	0.361	0.423
(13) Use a nutrition information panel to make food choices	0.178	0.210	**0.400**
(14) Check the use by date on foods ^1^	0.148	0.213	0.299
(15) Run out of money for food	0.325	0.126	–0.042

^1^ Question not included in Version 1 of the questionnaire. The questionnaire items are listed in the leftmost column.

## Data Availability

The data that support the findings of this study are available from Department of Health, Western Australia, but restrictions apply to the availability of these data, which were used under license for the current study and so are not publicly available. Data are, however, available from the authors upon reasonable request and with permission of Foodbank WA and Department of Health, Western Australia.
